# Validity and Reliability of Intraoral Camera with Fluorescent Aids for Oral Potentially Malignant Disorders Screening in Teledentistry

**DOI:** 10.1155/2021/6814027

**Published:** 2021-10-29

**Authors:** Sirikanlaya Vetchaporn, Wetchayan Rangsri, Jitjiroj Ittichaicharoen, Pimduen Rungsiyakull

**Affiliations:** ^1^Division of Geriatric Dentistry, Faculty of Dentistry, Chiang Mai University, Chiang Mai 50200, Thailand; ^2^Intercountry Centre for Oral Health, Department of Health, Ministry of Public Health, Chiang Mai 50000, Thailand; ^3^Department of Mechanical Engineering, Faculty of Engineering, Chiang Mai University, Chiang Mai 50200, Thailand; ^4^Department of Oral Biology and Oral Diagnostic Sciences, Faculty of Dentistry, Chiang Mai University, Chiang Mai 50200, Thailand; ^5^Department of Prosthodontics, Faculty of Dentistry, Chiang Mai University, Chiang Mai 50200, Thailand

## Abstract

There is limited documentation of using fluorescence images in oral potentially malignant disorders (OPMDs) and oral cancer screening through the field of teledentistry. This study aims to develop and evaluate the validity and reliability of the intraoral camera with the combination method of autofluorescence and LED white light used for OPMDs and oral cancer screening in teledentistry. The intraoral camera with fluorescent aids, which uses a combined method of both autofluorescence and LED white light, was developed before the device was evaluated for validity and reliability as a OPMDs screening tool for teledentistry. All lesions of thirty-four OPMD patients underwent biopsy for definitive diagnosis and were examined by an oral medicine specialist. Both images under autofluorescent and LED white light mode captured from the device were sent online and interpreted for the initial diagnosis and dysplastic features in addition to being compared to the direct clinical examination and histopathological findings. The combination method was also compared with autofluorescence method alone. The device provided good image quality, which was enough for initial diagnosis. Using the combination method, sensitivity, specificity, PPV, and NPV of the device via teledentistry were 87.5%, 84.6%, 63.6%, and 95.7%, respectively, which were higher than autofluorescence method alone in every parameter. The concordance of dysplastic lesion was 85.29% and 79.41% for category of lesion. The validity and reliability results of the combination method for the screening of dysplasia in OPMDs were higher than autofluorescent method alone. The intraoral camera with fluorescent aids for the OPMDs screening can be utilized for screening via teledentistry.

## 1. Introduction

Ninety percent of squamous cell carcinomas (SCC) are developed from oral potentially malignant disorders (OPMDs) [1]. OPMDs progress from hyperplasia to an increasing degree of dysplasia and finally into carcinoma in situ [2]. Autofluorescence is one of the diagnostic aids for screening detection of OPMDs and oral cancer. The principle is to illuminate the oral tissue with UV and blue excitation light [3–5]. The beams enter the tissue and are absorbed by “the fluorophores molecules”; then the fluorophores reemit the specific wavelength of fluorescence, which is a longer wavelength in the almost green, yellow, and red color spectrum [3, 4]. The various spectrums used as an excitation and emission wavelength are dependent on the equipment systems or the commercial product available in those studies [3–6]. For example, VELscope™ is a direct visualization tissue fluorescence device recognized by the WHO in 2009 as a commercialized medical device that produces 400–460 nm of excitation spectrum [4, 6].

The dysplastic detection in OPMDs leads to timely referral and treatment [7, 8]. Teledentistry could improve access to oral healthcare while also having the benefit in early intervention, oral health education, and effectiveness of oral health services. The image tools widely used in teledentistry are digital cameras, smartphone cameras, and intraoral cameras, which are usually used in the field of general dentistry and pediatric dentistry as caries detection. Previous studies have shown that the validity and reliability of those devices used as teledentistry tools could be comparable to conventional direct examination for oral screening [9–11]. However, there is limited documentation and investigation on the validity and reliability of using fluorescence images from an autofluorescence method in teledentistry through the field of oral medicine for OPMDs and oral cancer screening as previous studies used only images under normal white light to evaluate via teledentistry [10, 11]; fluorescence images were not yet been included. The combination method between autofluorescence and white light from light-emitting diodes (LED) could be equivalent to a conventional oral examination and autofluorescence method may increase validity for detection of epithelial dysplasia.

The purposes of this study were to develop and evaluate the validity and reliability of the intraoral camera with fluorescent aids, which use a combination method of both autofluorescence and LED white light to screen OPMDs in teledentistry. The validity and reliability were also compared with autofluorescence method alone.

## 2. Materials and Methods

### 2.1. Devices

The intraoral camera with fluorescent aids for the screening of OPMD in teledentistry consists of 5 main components:

1. UV-blue light source (Marubeni, USA): a light source utilizing 10 UV LEDs, which provides an excitation spectrum composed primarily of the 360–450 nm (UV-A) wavelength light.
2. White light source (Inskam, China): conventional LED were used as white light source. Four of them were coupled around the camera on the tip of the handheld device. White light and UV-blue light could be alternated for use either in LED white light mode or in autofluorescent mode.
3. Camera image sensor and lens (Inskam, China): CMOS image sensor came with the maximum camera resolution of 2594 × 1944P, 5 million pixels. The diameter of camera lens is 6 mm with IP68 waterproof grade with a lens system that provides autofocus of the focal length between 2 centimeters and infinity (*f* = 2−∞).
4. Light filter (Knight Optical, UK): a 480 nm long-pass filter was used as a fluorescent light filter, allowing the reemission of fluorescent wavelength from 480 nm and above spectrum to pass.
5. Controller and processing units: these units were packed into a controller box that is responsible for controlling the intensity of the light and also syncing images or video received from camera to the smartphone via WiFi (built-in IPEX antenna with operating frequency of 2.4 GHz, IEEE 802.11 b/g/*n* network standard).

The device was used with a free application (Inskam, China) available on a smartphone or tablet for both iOS and android platforms. The images stored can be sent online via any application as electronic information for teledentistry. The camera image sensor and lens were mounted at the tip of the handle, arranged at 135-degree angulation to the handle axis base on “mouth mirror design.” The handle is connected to the controller and processing units which can control the intensity of the light and sync images or video received from the camera to the smartphone via WiFi. The UV to blue spectrum light of the devices is safe to use because its spectrum is the same as those used in composite curing light. Additionally, the devices light intensity is much less than the intensity of curing light. The intraoral camera with fluorescent aids used in OPMDs and oral cancer screening can emit the maximum irradiance of blue excitation light at about 2000 Lux, also 7,500 Lux for LED white light at the focal length of 2.5 cm from the tissue.

**Figure 1:**
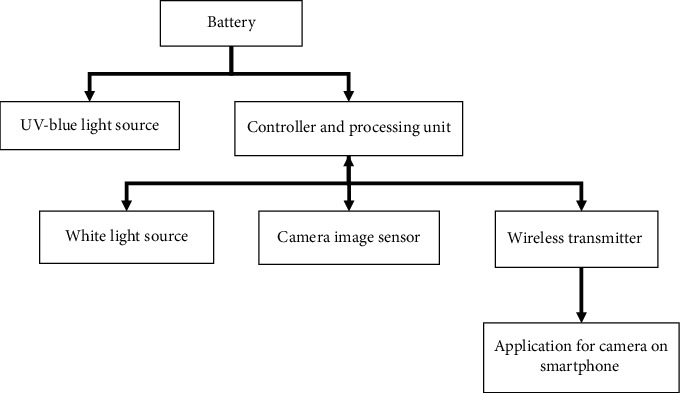
The overall device mechanism.

**Figure 2:**
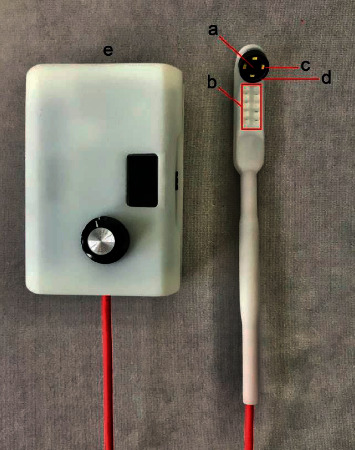
The intraoral camera with fluorescent aids for the screening of OPMDs used in teledentistry. This device consists of (a) camera image sensor and lens, (b) UV-blue light source, (c) white light source, (d) light filter, and (e) controller and processing units with rechargeable battery.

The device mechanism is shown in Figure 1. The intraoral camera with fluorescent aids for the screening of OPMDs used in teledentistry is shown in Figure 2.

**Figure 3:**
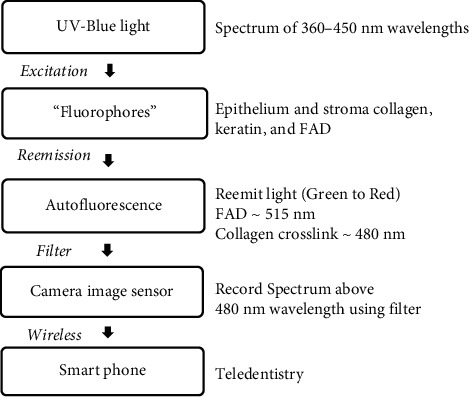
Devices mechanism for autofluorescent mode.

Under the autofluorescent mode, the principle is to illuminate oral tissue with an appropriate light source that is mostly in the UV to blue range of the spectrum. The excitation UV-blue light spectrum of 360–450 nm wavelength from 10 UV LEDs was used to stimulate fluorophores molecules in the epithelium and stroma. The fluorophores molecules then reemitted the fluorescence in several wavelengths. A 480 nm long-pass light filter was used to filter the reemission of fluorescent wavelength from 480 nm and above spectrum into the camera image sensor. Thus the camera can detect the green to red fluorescent light, while the blue excitation light is rejected. The light filter is removable from the front of the small camera, which allows switching between LED white light mode and autofluorescent mode. The device mechanism in autofluorescent mode is shown in Figure 3.

### 2.2. Patient Recruitment

An ethical approval was obtained from the Faculty of Dentistry Human Experimentation Committee (approval no. 80/2020), Chiang Mai University. Patients who had signed the informed consent documents were recruited from the Oral Biology and Oral Diagnosis Clinic, Faculty of Dentistry, Chiang Mai University, from December 2020 to March 2021.

Inclusion criteria were patients aged above 20 years old (1) who have lesions of OPMDs, (2) who have squamous cell carcinoma (SCC), (3) who permitted oral photography using the intraoral camera devices, and (4) who could have a tissue biopsy under local anesthesia.

Exclusion criteria are patients with other inflammation lesions including traumatic or aphthous ulcer.

### 2.3. Sample Collection

**Figure 4:**
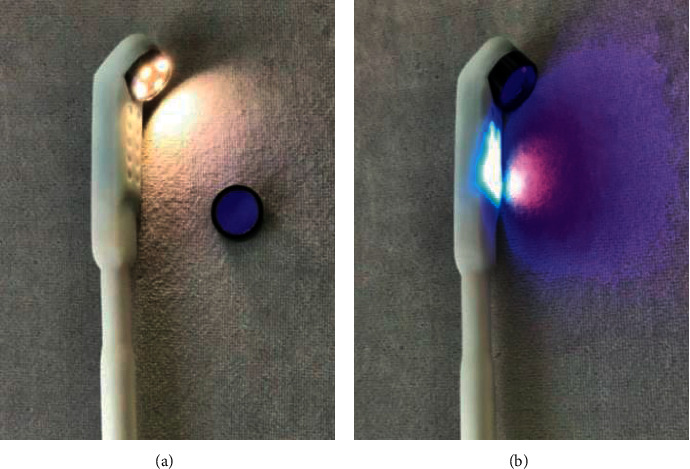
Application of the devices in (a) LED white light mode and (b) autofluorescent mode.

**Figure 5:**
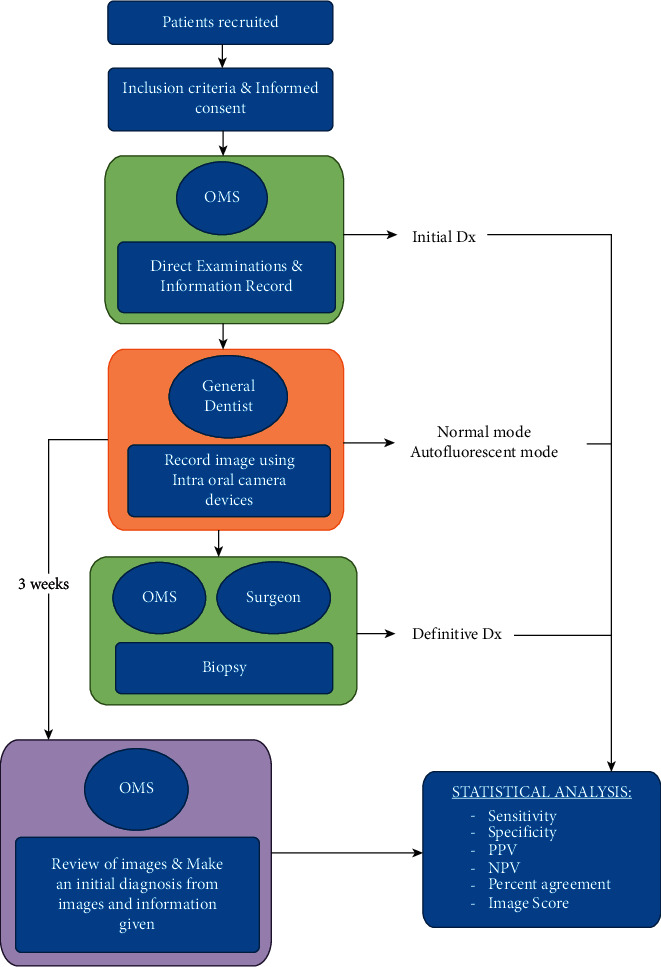
The study workflow.

The information of each patient including gender, age, hospital number, and subjective symptoms was required. Patients receiving an oral screening for OPMDs and oral cancer by an oral medicine specialist (OMS) were screened in seventeen locations of the oral cavity including (1) lips, (2) upper labial gingiva and vestibule, (3) upper left gingiva and vestibule, (4) upper right gingiva and vestibule, (5) lower labial gingiva and vestibule, (6) lower left gingiva and vestibule, (7) lower right gingiva and vestibule, (8) left buccal mucosa, (9) right buccal mucosa, (10) left retromolar area, (11) right retromolar area, (12) hard and soft palate, (13) dorsal tongue, (14) left lateral tongue, (15) right lateral tongue, (16) ventral tongue, and (17) floor of mouth. Each location of each patient was coded as a number. The patient information and initial diagnosis of the lesions were noted in the examination forms. In the same visit, a general dentist took an image of the most severe lesion of each patient using the intraoral camera in both LED white light mode and autofluorescent mode. Application of the intraoral camera with fluorescent aids for the screening of OPMDs in each mode was shown in Figure 4. All patients underwent tissue biopsy under a local anesthesia by the OMS or the oral surgeon. As it was known, some OPMDs have more than one histopathological feature in one lesion. The site of biopsy was chosen at the most severe features of the lesions to search for the worst diagnosis that the lesion could be. If the lesion has many curious characteristic features, the surgeon also took more than one site of each different feature. The most severe diagnosis is then analyzed in the research results. The resolution of the intraoral camera used to capture images was full HD (1920 × 1080 pixels). Each image code number was matched with the code in the screening form. After the washout time period, the OMS then was asked to do the diagnosis again from fluorescent image and the intraoral images under LED white light. Signs and symptoms and other information were also given. The reexamination data from the devices sent online through teledentistry was used to compare with the conventional direct examination and histological data from biopsy results. The study workflow is shown in Figure 5.

### 2.4. Examiner and Interpreter

All patients were examined in a prospective manner by an oral medicine specialist (OMS) who had acquired a Diploma of the Thai Board of Oral Diagnostic Sciences. The intracalibration was done to ensure that the analyzed result did not engage with the examiner error. To ensure that interpreter was reliable, OMS as the interpreter was asked to diagnose the set of OPMDs images twice, one week apart each time. Then the result of diagnosis which was done in the first time was compared with the second time and analyzed for concordance as a percent agreement. Showing 82.35% of percent agreement for the concordance, thus the interpreter was reliable since the statistic shows strong concordance.

### 2.5. Data Analysis

There were 3 parameters of the data to be analyzed including (1) category of lesion, (2) dysplasticity of lesion, and (3) image score.

The parameter of category of lesion was the initial diagnosis by the OMS according to patient's signs and symptoms and a clinical characteristic of the lesion. The category of lesion acquired from conventional direct examination was then compared with the reexamination data of reviewed images from the devices with the same code number. The percent agreement statistic was used for evaluation of diagnostic concordance as reliability. The parameter of dysplasticity of lesion was judged by fluorescence loss or low intensity of green fluorescence in the fluorescence images. OMS determined whether the lesion was dysplastic or not using combination method of both fluorescent images and images under LED white light and also fluorescence image from autofluorescence method alone. The parameter of dysplasticity of lesion was also compared with the histopathological results from biopsy. For the parameter of image score, OMS graded each image with score 0 to 2. The information of the three parameters is shown in Table 1.

## 3. Results and Discussion

### 3.1. Sample Characteristic

The demographic data of 34 patients enrolled in this study is provided in Table 2. Most of the patients were aged 50–59 years old. According to conventional direct examination, 19 lesions were diagnosed as oral lichen planus, which were the most common lesions found for the parameter of category of lesion. Nine lesions were clinically diagnosed as leukoplakia, 5 lesions were clinically diagnosed as discoid lupus erythematosus, and only one lesion was clinically diagnosed as a squamous cell carcinoma. All of the lesions underwent surgical biopsy, revealing 8 lesions as premalignant mild epithelial dysplasia for the parameter of dysplasticity of lesion, while the histopathological diagnosis shows the definitive diagnosis including 18 lesions of lichen planus, 5 lesions of discoid lupus erythematosus, 1 lesion of verruca vulgaris, 2 lesions of hyperkeratosis, and 8 lesions of premalignant mild epithelial dysplasia.

### 3.2. Validity and Reliability of the Devices

The parameter of dysplasticity of lesion was compared with the histopathological results from biopsy as a gold standard. Sensitivity, specificity, PPV, and NPV of the device using combination method were 87.5%, 84.6%, 63.6%, and 95.7%, respectively, while the results were 50.0%, 80.8%, 44.4%, and 84.0%, respectively, for autofluorescence method alone (AF alone). Dysplasticity of lesion parameter acquired from combination method was 85.29% agreement while the concordance for AF alone was 73.53%. The concordance between clinical direct examination and images reviewing from the devices via teledentistry for determining the category of lesion on initial diagnosis was 79.41% agreement (Table 3).

### 3.3. Images Quality

OMS was accessed for all images by reviewing online via “Line Application” using the same smartphone (Apple Inc., USA), which would not alter the image resolution and image size. The median and mode value of image score were 1 (SD = 0.3937).

## 4. Discussion

**Figure 6:**
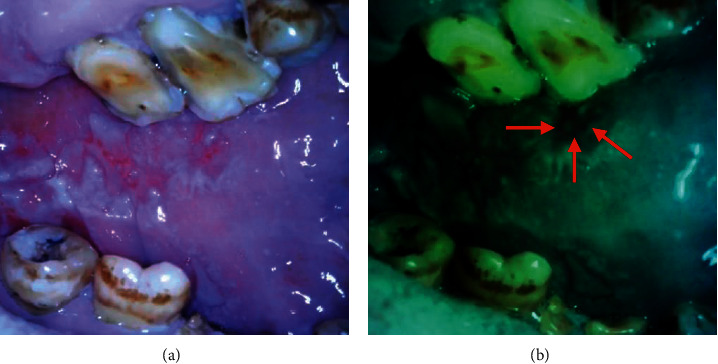
Intraoral images from the devices in (a) LED white light mode and (b) autofluorescent mode showing loss of fluorescence in suspicious dysplastic area.

**Figure 7:**
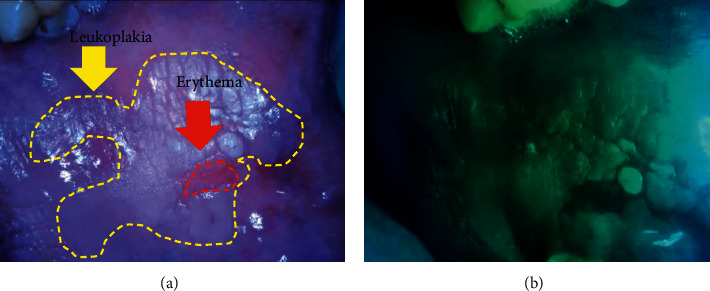
Images from the devices in (a) LED white light mode and (b) autofluorescent mode showing loss of fluorescence in erythema area. The image was in higher contrast, which could easily determine the outline of the lesion.

Since the dysplasticity of lesion could not be evaluated under LED white light image, autofluorescence is one of the diagnostic aids for screening detection of OPMDs and oral cancer. The validity and reliability of autofluorescence using in a direct fluorescence visualization device for the screening of epithelial dysplasia in OPMD and oral lesions have been assessed in previous studies [12, 13]. Several studies used only autofluorescence method alone while the others used a combination with a conventional oral examination [12, 14–17]. However those studies performed the examinations on site; teledentistry was not involved. The results are provided only in direct optical images and cannot be transferred as electronics information via teledentistry. The intraoral camera with fluorescent aids for OPMDs screening in teledentistry designed from this study could provide good image quality, which is enough to get the initial diagnosis. The image quality is important for making a decision for diagnosis, since previous study showed that the overall sensitivity and specificity of images used in teledentistry were dependent on image resolution to detect premalignant lesion and oral cancer [18]. Since the camera image sensor of the devices can produce high resolution images, up to 2K, the image resolution then depends on the resolution of the smartphone display when viewing images. Also, the application software for transferring the data through the Internet should not be the one that would reduce the image definition. Some images from the devices were shown in Figures 6 and 7. Another factor that could disturb the quality of the image is the light reflection, as light reflection could leave bright defects on the image, which could affect the interpretation of the lesions.

In this study, the device provides both LED white light mode and autofluorescent mode. This was equivalent to the same rationale used for the combination method, between conventional oral examination under LED white light and conventional direct fluorescence visualization device in autofluorescence method comparing to other studies. The systematic study showed that sensitivity and specificity of VELscope™, as an adjunctive tool to conventional oral examination for detection OPMD and/or SCC, were 73.9%–100% and 38%–97.9%, respectively [13]. Sensitivity, specificity, PPV, and NPV of the device using combination method were 87.5%, 84.6%, 63.6%, and 95.7%, which were higher than using autofluorescent method alone (50.0%, 80.8%, 44.4%, and 84.0%, respectively). From the results, low value of PPV on the parameter of dysplasticity of lesion might occur due to the low prevalence of dysplasia. There were only 23.53% (8 lesions) of all OPMDs samples that had mild dysplasia. The results showed that the value of percent agreement statistics for dysplasticity of lesion was 85.29%, which was strong and indicated that the device using combination method was a reliable tool in teledentistry. The results were consistent with other studies that revealed the combination method could improve the specificity [6, 12]. This might be because the images under LED white light mode can provide better clinical characteristics of the lesion than those in autofluorescence mode alone. These clinical characteristics could also help in diagnosis determination.

When interpreting fluorescence images acquired from the device, the loss of fluorescence, seen as dark areas from the device, was due to many factors. The alteration of abnormal dysplastic tissue is one of the situations that cause fluorescence loss. The presence of neoplasm, demonstrated by the different scattering and absorption properties of the light through the tissue, was dependent on the concentration of fluorophores that are found in the tissue matrix or in cells compositions such as flavin adenine dinucleotide (FAD), collagen, elastin, and keratin. Dysplastic tissue usually shows the loss of fluorescence, represented by a dark area under fluorescent light [3, 4]. The low intensity of fluorescence is the result of (1) the collagen breakdown following the invasion of dysplastic epithelial cells and (2) the increase in metabolic activities of dysplastic cells followed by the reduction of free FAD [3, 4]. Moreover, the increase in blood supply within the dysplastic lesion caused the accumulation of hemoglobin, which strongly absorbs blue and green light; thus the less reemission of fluorescence intensity was seen [3, 4].

The loss of fluorescence must be always considered with the clinical findings or signs and symptoms of the patient [3, 4]. Since the fluorescence image results do not yield a “yes or no answer,” the device cannot be a replacement for definitive diagnosis. The device can be used as a clinical adjunct or screening tool rather than a diagnostic tool. It is more helpful to outline a determination before any biopsy for the histological evaluation since it can provide higher contrast between abnormal lesion and surrounding normal tissue. The interpretation of fluorescence loss could affect validity of the device, since darkness of the fluorescence loss in images is judged by human perceptual skill, which is very varying. On the other hand, artificial intelligence (Ai) could be the solution of these limitations, as they allow image acquisition, feature extraction, and mathematical analysis and demonstrate them as objective values [19–21]. Moreover, fluorescence images acquired from the device can be used for developing an artificial intelligence (Ai) diagnosis system as a measure for mass populations screening.

In practicality, the intraoral camera with fluorescent aids for the screening of OPMDs is portable and suitable for use as teledentistry tools in primary healthcare units in the community. In particular, the device is practical for dependent older adults, who have limitations in mouth opening. The intraoral part of the devices is small and can be performed in a dark environment due to its own light sources. Another advantage is that it is more ergonomic than the conventional direct fluorescence device, as dentists do not need to bend forward close to the patient mouth and star into the loupes. The intraoral image or fluorescence image is shown clearly on the display of smartphone in real time and can be transmitted as electronic information in teledentistry via the Internet. The device properties and characteristics are shown in Tables 4 and 5, respectively.

## 5. Conclusions

The intraoral camera with fluorescent aids, which has the combined advantages of both conventional intraoral camera and conventional direct fluorescence device, can be utilized as an adjunctive device for screening of dysplasia in OPMDs via teledentistry. According to the combination method between autofluorescence and an examination under LED white light, validity and reliability for the screening of dysplasia in OPMDs were higher than autofluorescence method alone. It is important that the results from device utilizing optical fluorescence imaging should be interpreted with the clinical findings or signs and symptoms of the patient. While the conventional intraoral camera cannot assess a parameter of dysplastic of the lesions, the intraoral camera with florescent aids can be a more useful tool in screening of dysplasia in OPMDs. The application on smartphone using with the intraoral camera in teledentistry should not alter the images quality.

## Figures and Tables

**Table 1:** Parameters of the data.

Category of lesion	Dysplasticity of lesion	Image score
(i) Leukoplakia	(i) Dysplasia	0
(ii) Erythroplakia	(ii) No dysplasia	(i) Image quality is poor
(iii) Lichen planus (LP)	—	(ii) Not enough to get a diagnosis
(iv) Discoid lupus erythematosus (DLE)	—	1
(v) Palatal lesions in reverse smokers	—	(i) Image quality is fair
(vi) Oral submucous fibrosis (OSMF)	—	(ii) Enough to get a diagnosis
(vii) Actinic cheilitis	—	2
(viii) Squamous cell carcinoma (SCC)	—	(i) Image quality is good
—	—	(ii) Enough to get a diagnosis

**Table 2:** Demographic data of patients examined.

	*n* (%)
Gender	
Male	16 (47.06)
Female	18 (52.94)

Age	
20–29	1 (2.94)
30–39	1 (2.94)
40–49	3 (8.82)
50–59	13 (38.24)
60–69	9 (26.47)
70–79	7 (20.59)

Category of lesion (initial Dx by OMF)	
Leukoplakia	9 (26.47)
OLP	19 (55.88)
DLE	5 (14.71)
SCC	1 (2.94)

Histopathological diagnosis	
OLP	18 (52.94)
DLE	5 (14.71)
Verruca vulgaris	1 (2.94)
Hyperkeratosis	2 (5.88)
Mild epithelial dysplasia	8 (23.53)

Dysplasticity of lesion (histopathologic result)	
Dysplasia	8 (23.53)
No dysplasia	26 (76.47)

Dysplasticity of lesion (combination method)	
Dysplasia	11 (32.35)
No dysplasia	23 (67.65)

Dysplasticity of lesion (AF alone)	
Dysplasia	9 (26.47)
No dysplasia	25 (73.53)

Image score	
0	0 (0.00)
1	29 (85.29)
2	5 (14.71)

**Table 3:** Validity and reliability of the devices.

	AF alone	Combination method (AF + white LED)
Sensitivity	50.0% (15.3–84.7%)	87.5% (64.6–110.4%)
Specificity	80.8% (65.6–95.9%)	84.6% (70.7–98.5%)
PPV	44.4% (11.9–76.9%)	63.6% (35.2–92.1%)
NPV	84.0% (69.6–98.4%)	95.7% (87.3–103.9%)
Percent concordance (dysplasticity of lesion)	73.53%	85.29%
Percent agreement (category of lesions)	N/A	79.41%

**Table 4:** The intraoral camera with fluorescent aids for the screening of OPMDs properties.

Device properties	Conventional intraoral camera	Conventional direct fluorescence visualization device	Device developed from the study
Capturing and recording intraoral images/videos	✓	—	✓
Small size and angulation design based on “mouth mirror design”	—	—	✓
LED white light mode	✓	—	✓
Blue light mode	✓/—	✓	✓
Autofluorescent mode (i) Identifying oral potentially malignant disorders and oral cancer (ii) Identifying poor oral hygiene due to bacterial aggregation	—	✓	✓
Data sync via smartphone	✓/—	—	✓

**Table 5:** Characteristics of the intraoral camera with fluorescent aids for the screening of OPMDs.

Strengths	Limitations
1. Noninvasive	1. Image results should be interpreted by experienced clinicians
2. Portable and simple to use	2. Need a dark environment in autofluorescent mode
3. Provide both real-time and recorded images/videos	—
4. Cost-effective and no consumable reagents cost	—
5. Can be performed by wide range of operators after short training	—
6. Useful in teledentistry field	—

## Data Availability

The (pdf) data used to support the findings of this study are available from the corresponding author upon request.
